# Weight at birth and adolescence and premenopausal breast cancer risk in a low-risk population

**DOI:** 10.1038/sj.bjc.6600009

**Published:** 2002-01-07

**Authors:** M Sanderson, X O Shu, F Jin, Q Dai, Z Ruan, Y-T Gao, W Zheng

**Affiliations:** University of Texas School of Public Health at Brownsville, University of Texas Brownsville, 80 Fort Brown, Brownsville, Texas, TX 78520, USA; Center for Health Services Research and Vanderbilt-Ingram Cancer Center, Vanderbilt University, Nashville, Tennessee, TN 37232-8300, USA; Department of Epidemiology, Shanghai Cancer Institute, Shanghai, People's Republic of China

**Keywords:** breast cancer, birth weight, adolescent weight, adult body size

## Abstract

We assessed breast cancer risk in relation to weight at birth and adolescence. In-person interviews were completed with the biological mothers of women aged 45 years and younger who participated in the Shanghai Breast Cancer Study in 1996–98 (288 cases, 350 controls). After adjustment for confounding, women who were 4000 g or more at birth were not at increased risk of breast cancer (odds ratio=0.7; 95% confidence interval 0.4–1.4) relative to women whose birth weight was 2500–2999 g. Compared with women of average perceived weight at age 15 years, no relation was apparent for heavier than average weight based on maternal report (odds ratio=0.7; 95% confidence interval 0.5–1.2) or self-report (odds ratio=1.0; 95% confidence interval 0.7–1.6). Perceived adolescent weight and height did not modify the association of birth weight with breast cancer risk. These results suggest that weight early in life is not related to premenopausal breast cancer risk in this low-risk population.

*British Journal of Cancer* (2002) **86**, 84–88. DOI: 10.1038/sj/bjc/6600009 www.bjcancer.com

© 2002 The Cancer Research Campaign

## 

Premenopausal breast cancer has been linked to high birth weight (Ekbom *et al*, 1992; Innes *et al*, 2000; Michels *et al*, 1996; Sanderson *et al*, 1996). Conversely, high adolescent (Coates *et al*, 1999; Hislop *et al*, 1986; Le Marchand *et al*, 1988a), early adult (Coates *et al*, 1999; Huang *et al*, 1997; Trentham-Dietz *et al*, 1997) and adult weight or body mass index (Brinton and Swanson, 1992; Huang *et al*, 1997; Swanson *et al*, 1996; Ursin *et al*, 1995; van den Brandt *et al*, 2000) appear to be protective against premenopausal breast cancer. Several studies have investigated the association between breast cancer and weight at birth (De Stavola *et al*, 2000; Ekbom *et al*, 1992, 1997; Innes *et al*, 2000; Le Marchand *et al*, 1988b; Michels *et al*, 1996; Sanderson *et al*, 1996, 1998a) or weight at adolescence (Brinton and Swanson, 1992; Choi *et al*, 1978; Coates *et al*, 1999; Franceschi *et al*, 1996; Hislop *et al*, 1986; Le Marchand *et al*, 1988a; Pryor *et al*, 1989) with inconsistent findings. Possible limitations of these studies related to exposure measurement and age at diagnosis of breast cancer.

Since self-report of body size in early life is prone to misclassification, maternal report may be less subjective. Maternal report was available for two of the studies investigating breast cancer risk associated with birth weight (Michels *et al*, 1996; Sanderson *et al*, 1998a), but none of the studies of adolescent weight. The present analysis was conducted to assess whether birth weight and adolescent weight as reported by subjects' mothers were related to premenopausal breast cancer risk. In addition, we investigated whether perceived adolescent weight and height modified the association of birth weight with breast cancer risk.

## MATERIALS AND METHODS

Detailed methods of this population-based case–control study appear elsewhere (Gao *et al*, 2000). Briefly, all women aged 25–64 years who were permanent residents of urban Shanghai at the time of diagnosis of first primary invasive breast cancer (August 1996 through March 1998) were eligible for the study. Two senior pathologists histologically confirmed all diagnoses. We used rapid case ascertainment supplemented by the Shanghai Cancer Registry to identify breast cancer cases who had no prior history of cancer. A total of 1459 breast cancer cases (91.1% of eligible cases) completed a standardized in-person interview. Of potentially eligible cases, 109 refused (6.8%), 17 died prior to the interview (1.1%), and 17 were not located (1.1%).

The Shanghai Resident Registry, a listing of all permanent adult residents of urban Shanghai, was used to randomly select controls. Controls were frequency matched to cases on age (5-year interval) based on the number of incident breast cancer cases by age group reported to the Shanghai Cancer Registry from 1990 through 1993. Women who did not reside at the registered address at the time of the study were ineligible. A total of 1556 controls (90.4% of eligible controls) completed a standardized in-person interview. The remaining 166 potentially eligible controls (9.6%) refused to participate. Two women died prior to the interview and were excluded.

The study was approved by relevant institutional review boards in Shanghai and the United States. Women were interviewed at hospitals (cases) or at home (cases and controls) by trained interviewers. The subject questionnaire collected information on demographic factors, reproductive and medical histories, family history of cancer, use of oral contraceptives and hormone replacement therapy, diet, physical activity, lifestyle factors, and adolescent and adult body size. Women were asked how their perceived weight and height compared with their peers at the ages of 10, 15 and 20. After completing the interview, women were weighed and had their standing and sitting height, and waist and hip circumferences measured. Information on exposures pertained to the period before an assigned reference date, the diagnosis date for breast cancer cases and a similar date for controls.

The biological mothers of women the age of 45 and younger who resided in Shanghai provided detailed information about the subject's adolescent diet and body size, and about her pregnancy with the subject. In-person interviews were completed with the mothers of 296 cases and 359 controls (with respective response rates of 79.6 and 81.8%). Eight cases and nine controls were subsequently excluded because they were postmenopausal, resulting in 288 cases and 350 controls for this analysis.

We used unconditional logistic regression to estimate the relative risk of breast cancer associated with weight at birth and adolescence while controlling for confounders (Breslow and Day, 1980). All variables were entered into models as dummy variables. In multiple logistic regression models, we assessed linear trend by treating categorical variables as continuous variables.

## RESULTS

Table 1

**Table 1:**
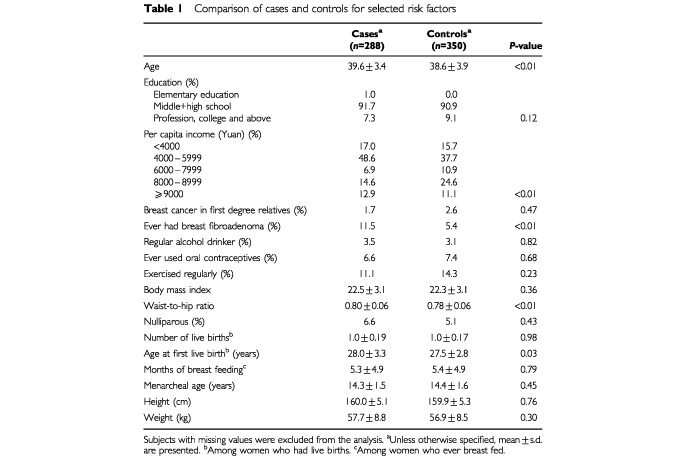
Comparison of cases and controls for selected risk factors

compares known breast cancer risk factors of cases and controls. Compared to controls breast cancer cases were slightly older, had a lower income, and were more likely to have a history of fibroadenoma, a higher waist-to-hip ratio, and a later age at first birth. For consistency with most previous studies, subsequent analyses were adjusted for family history of breast cancer, menarcheal age, parity, and all of the preceding variables, except waist-to-hip ratio. Since adult waist-to-hip ratio may be in the causal pathway between birth and adolescent weight and breast cancer, it and adult body mass index were assessed as effect modifiers rather than as confounders. Further adjustment of birth weight for other perinatal factors did not materially change the odds ratios. Perceived weight is adjusted for perceived height at specific ages and vice versa.

Table 2

**Table 2:**
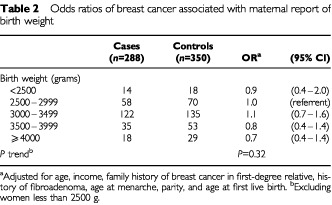
Odds ratios of breast cancer associated with maternal report of birth weight

presents the odds ratios (ORs) and 95% confidence intervals (CIs) for breast cancer associated with maternal report of birth weight. After adjustment for confounding factors, women who were 4000 g or more at birth were not at increased risk of breast cancer (OR=0.7; 95% CI 0.4–1.4) relative to women whose birth weight was 2500–2999 g. When we dichotomized birth weight an identical odds ratio for women whose birth weight was 3500 g or more (OR=0.7, 95% CI 0.5–1.1) was found, compared with women who were less than 3500 g.

The risks for breast cancer associated with maternal and subject perceptions of subjects' weight and height at the age of 15 separately and combined are shown in Table 3

**Table 3:**
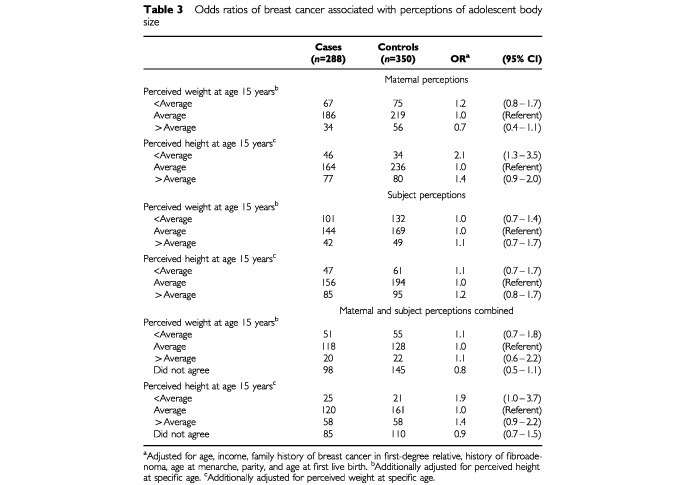
Odds ratios of breast cancer associated with perceptions of adolescent body size

. For mothers and subjects whose perceptions differed we created a fourth category. Compared with women of average perceived weight at the age of 15, no relation was apparent for heavier than average weight based on maternal report (OR=0.7; 95% CI 0.4–1.1) or self-report/combined maternal and subject report (OR=1.1; 95% CI 0.6–2.2). Elevated risks of breast cancer were seen for women whose mothers perceived they were shorter than average at age 15 (OR=2.1, 95% CI 1.3–3.5), which was reflected in the combined maternal and subject estimate (OR=1.9, 95% CI 1.0–3.7). We calculated Spearman correlation coefficients to assess the reliability of reporting of perceptions of weight and height by case–control status (Armstrong *et al*, 1992). The correlations comparing maternal and subject perceptions were reasonably consistent (weight *r*=0.46, height *r*=0.59).

Table 4

**Table 4:**
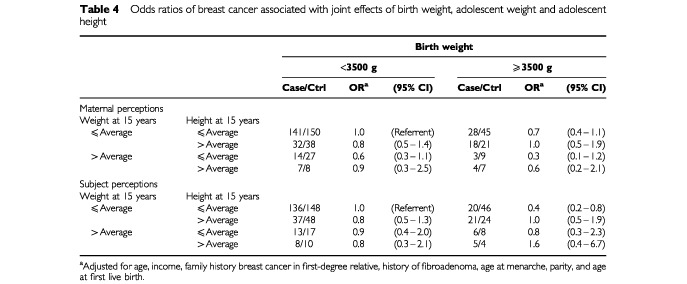
Odds ratios of breast cancer associated with joint effects of birth weight, adolescent weight and adolescent height

shows the joint effect of birth weight, adolescent weight, and adolescent height on breast cancer risk. The referent group is women who were less than 3500 g at birth, and who at the age of 15 were of average weight and average height. Perceived adolescent weight and height did not modify the effect of birth weight on breast cancer risk or vice versa. Women whose birth weight was 3500 g or more and who perceived themselves to be of low or average adolescent weight and low or average adolescent height were at reduced risk of breast cancer (OR=0.4, 95% CI 0.2–0.8). Neither adult body mass index nor waist-to-hip ratio modified the effect of birth weight or adolescent weight on breast cancer risk (data not shown).

## DISCUSSION

We found no association between high birth weight and premenopausal breast cancer, in agreement with some (De Stavola *et al*, 2000; Ekbom *et al*, 1997; Le Marchand *et al*, 1988b; Sanderson *et al*, 1998a), but not all (Ekbom *et al*, 1992; Innes *et al*, 2000; Michels *et al*, 1996; Sanderson *et al*, 1996), of the previous studies of this topic. Trichopoulos (1990) hypothesized that exposure to high levels of endogenous estrogen *in utero* may be a possible risk factor for subsequent breast cancer. In a study conducted in Greece, high birth weight was associated with high pregnancy estrogen levels (Petridou *et al*, 1990). However, Lipworth *et al* (1999) reported substantially higher mean levels of pregnancy estrogens and significantly lower mean birth weights among women in Shanghai than among their counterparts in Boston. They speculated that higher albumin and sex hormone binding globulin among Chinese women could decrease the bioavailability of oestrogens. This may partially explain the lack of a positive association with high birth weight observed in the present analysis.

The results of studies on adolescent weight and premenopausal breast cancer risk are inconsistent. Premenopausal breast cancer risk associated with heavier than average weight at the age of 15 or thereabouts was decreased in some studies (Coates *et al*, 1999; Hislop *et al*, 1986; Le Marchand *et al*, 1988a), increased in one study (Pryor *et al*, 1989), and had no association in other studies (Brinton and Swanson, 1992; Choi *et al*, 1978; Franceschi *et al*, 1996). The reduction in risk reported by Le Marchand *et al* (1988a) was for the highest tertile of body mass index compared with the lowest tertile (OR=0.45, 95% CI 0.23–0.86). This relation was more pronounced among women who were heavier than average during adolescence and whose adult body mass index was at or above the median (OR=0.31, 95% CI 0.16–0.60). In the present analysis, no relation was apparent for breast cancer associated with heavier than average perceived weight at the age of 15 based on maternal report or self-report. Neither adult body mass index nor waist-to-hip ratio modified the effect of perceived adolescent weight on breast cancer risk.

The biological mechanism that Stoll (1998) proposed to help explain the reduced risk of premenopausal breast cancer associated with adolescent obesity in some studies was that obesity triggered a hyperinsulinemic insulin resistance at puberty that could lead to abnormal ovarian steroidogenesis and anovulation. Most of the women in this study grew up during a period when food and meat were rationed and adolescent obesity was rare, thus perceived weight at the age of 15 may not reflect adolescent obesity as defined among Western women. Spearman correlation coefficients were calculated to assess whether age at menarche, used as a marker of adolescence, was correlated with perceived weight or height at the age of 15. Whether reported by the subject or her mother, these correlations were negative and clustered around zero.

In a previous analysis of this study, premenopausal breast cancer was unrelated to early adult and adult weight, but was associated with a high adult waist-to-hip ratio, even after adjustment for body mass index (Shu *et al*, 2001). These findings differ from the majority of studies of this topic conducted among Western women. As was the case for early adult and adult weight, an alternative explanation for the null associations found for weight at birth and adolescence and breast cancer risk is the paucity of women at the extremes of these measures.

Our findings of increased risks of premenopausal breast cancer associated with maternal report and combined maternal and subject report of perceived height as shorter than average at the age of 15 differs from all previous studies. Coates *et al* (1999) reported reduced risks for women who were much shorter than average at the ages of 15 to 16. Brinton and Swanson (1992) reported an increased premenopausal breast cancer risk associated with taller than average perceived height at the age of 16. An earlier adolescent growth spurt and tallness in childhood has been linked to earlier menarche (Preece, 1989), an established breast cancer risk factor. In the present study, the mean menarcheal age was approximately 14.5 years, which was nearly 2 years later than the mean age among US women at the time the majority of women in this study were achieving menarche (Zacharias *et al*, 1976). The later age at menarche experienced by women in China meant that some of the women in the present analysis had not undergone their adolescent growth spurt by the age of 15, which may partially explain the lack of a positive association observed in this study with taller adolescent height.

One previous study has investigated the joint effect of birth weight and adolescent weight or adolescent height on breast cancer risk. De Stavola *et al* (2000) recently examined the effects of birth weight and childhood growth on subsequent breast cancer risk in a cohort study in the UK. They reported a borderline increase in risk of premenopausal breast cancer associated with a birth weight of 3500 g or more (relative risk [RR]=2.31, 95% CI 0.93–5.74). This risk was modified by height at the age of 7, with no association among women who were short or average (RR=1.23, 95% CI 0.31–4.91) and a pronounced elevation in risk among women who were tall (RR=5.86, 95% CI 1.97–17.44). They concluded that the birth weight and breast cancer relation might be mediated through childhood growth. Height at the age of 7 was chosen to reflect pre-pubertal growth, but there was no significant interaction for the height at the age of 15. In the present analysis, perceived height at the age of 10 (data not shown) and the age of 15 did not modify the effect of birth weight on breast cancer risk. However, women who were 3500 g or more and short or average height at the age of 15 were at decreased risk of breast cancer.

There were several limitations of this study. Data on birth weight and maternal perception of adolescent body size analyses were available only in a subgroup of premenopausal women, reducing statistical power to detect effect modification. The narrow distribution of weights at birth and adolescence in China (Eveleth and Tanner, 1976; Fung *et al*, 1989) may have further limited the statistical power to evaluate the association of these variables with breast cancer risk. Reporting of birth weight and perceptions of weight and height during adolescence are prone to misclassification. However, in a study conducted in Washington State, we found very high correlations between maternal reporting and birth certificate recording of birth weight (case mothers *r*=0.89, control mothers *r*=0.84) (Sanderson *et al*, 1998b). To our knowledge, no validation studies of maternal reporting of adolescent body size have been conducted.

This study has many strengths. The population-based nature of the study and its high response rates among subjects (cases: 91%; controls: 90%) and their mothers (case mothers: 80%; control mothers: 82%) minimizes selection bias. We adjusted for known breast cancer risk factors, and evaluated the weight at birth and adolescence and breast cancer associations in conjunction with suspected effect modifiers of these relations. An additional strength of the study was the good agreement between maternal and subject reporting of adolescent body size. There are, however, some measurement errors, which may have attenuated the estimated odds ratios is this study.

In summary, our study indicates that weight at birth and adolescence has little influence on breast cancer risk in Chinese women. These results suggest that weight early in life is not related to premenopausal breast cancer risk in this low-risk population. Future studies should assess these relations to clarify the role that weight early in life may play in breast cancer risk.
